# Tratamiento con dabrafenib y trametinib en un caso de colangiocarcinoma intrahepático con mutación BRAF V600E

**DOI:** 10.23938/ASSN.1159

**Published:** 2026-06-24

**Authors:** Arturo Gámiz Rejano, Ana Isabel Gago Sánchez, Rosa María Rodríguez Alonso

**Affiliations:** 1 Servicio de Farmacia Hospitalaria Hospital Universitario Reina Sofía Córdoba España; 2 Servicio de Oncología Médica Hospital Universitario Reina Sofía Córdoba España

**Keywords:** Colangiocarcinoma, BRAF V600E, Dabrafenib, Trametinib, Terapia Dirigida, Intrahepatic Cholangiocarcinoma, BRAF V600E, Dabrafenib, Trametinib, Targeted Therapy

## Abstract

El colangiocarcinoma intrahepático es una neoplasia de mal pronóstico con opciones terapéuticas limitadas en estadios avanzados. La identificación de alteraciones moleculares accionables permite considerar terapias dirigidas en subgrupos seleccionados.

Presentamos nuestra experiencia clínica con un varón de 74 años con colangiocarcinoma intrahepático metastásico con mutación BRAF V600E, tratado fuera de indicación en segunda línea con dabrafenib (150 mg/12 h) y trametinib (2 mg/24 h) tras progresión a quimioterapia basada en platino. El tratamiento se asoció a enfermedad estable, descenso de CA 19-9 y toxicidad leve, logrando una supervivencia libre de progresión de 12,8 meses y una supervivencia global de 44,4 meses. Este caso refuerza la utilidad de integrar de forma temprana la caracterización molecular, así como el valor de la terapia dirigida en práctica clínica en tumores de baja supervivencia y con escasas alternativas farmacológicas.

## Abstract (en)

Intrahepatic cholangiocarcinoma is a poor prognosis malignancy with limited therapeutic options in advanced stages. Identification of actionable molecular alterations may enable targeted therapies in selected patients.

We describe a 74-year-old man with metastatic intra-hepatic cholangiocarcinoma harboring a BRAF V600E mutation, treated off-label in the second-line setting with dabrafenib (150 mg twice daily) plus trametinib (2 mg once daily) after progression on platinum-based chemotherapy. Treatment resulted in stable disease, a reduction in CA 19-9, and mild adverse events. Progression-free survival was 12.8 months and overall survival reached 44.4 months. This case underscores the importance of early molecular profiling and supports the integration of targeted therapies into routine clinical practice for tumors with limited effective therapeutic options.

## INTRODUCCIÓN

El colangiocarcinoma (CCA) es una neoplasia maligna caracterizada por una amplia heterogeneidad fisiopatológica, tumoral y molecular. Según su localización anatómica se clasifica en intrahepático (menos frecuente, 10-20% de los casos) y extrahepático, con dos subtipos: el más frecuente es el CCA perihiliar (pCCA), que representa aproximadamente el 50-60% de los casos, mientras que el distal (dCCA) supone el 20-30%^1^.

El CCA representa el 15% de los tumores hepáticos primarios y el 3% de los gastrointestinales^1^. En el 70% de pacientes, su diagnóstico en etapas avanzadas limita las opciones terapéuticas y se asocia a un pronóstico desfavorable con una frecuencia de recidiva entre el 42 y el 70%, una supervivencia del 7-20% a 5 años y 28 meses de mediana de supervivencia^1^^,^^2^.

Las opciones de tratamiento de primera línea para tumores localmente avanzados, irresecables o metastásicos, son quimioterapia con gemcitabina y cisplatino con durvalumab o pembrolizumab. En segunda línea se emplean regímenes con ácido folínico, 5 fluorouracilo y oxaliplatino (FOLFOX), 5-fluorouracilo y oxaliplatino (FUOX) o 5-fluorouracilo con o sin irinotecán, con una eficacia modesta y toxicidad significativa^2^^-^^7^.

Mediante secuenciación de nueva generación **(**NGS**)** se han descrito alteraciones genéticas en el CCA^5^ con diferente perfil mutacional según la localización del tumor primario; las mutaciones dirigibles se encuentran con mayor frecuencia en CCA intrahepáticos. Las alteraciones genómicas dirigibles con mayor prevalencia son mutaciones IDH1 (20%), fusiones FGFR2 (15%), amplificaciones ERB2 (10%), mutaciones PIK3CA (7%) y mutación BRAF V600E (5%), entre otras^8^.

La mutación BRAF V600E consiste en la sustitución de la valina por un ácido glutámico en el codón 600 del gen BRAF y afecta a la proteína quinasa BRAF que forma parte de la vía RAS-RAF-MEK-ERK involucrada en proliferación, migración y supervivencia celular^9^. Trametinib y dabrafenib inhiben dos quinasas de la misma vía, MEK y BRAF, proporcionando una doble inhibición^3^.

Dabrafenib con trametinib está indicado por la Agencia Europea de Medicamentos (EMA) y por la Agencia Española de Medicamento y Productos Sanitarios (AEMPS) exclusivamente para pacientes con mutación BRAF V600E en melanoma metastásico o irresecable, en el tratamiento adyuvante del melanoma tras resección completa y en cáncer de pulmón no microcítico avanzado^10^^,^^11^.

Presentamos este caso de un paciente con colangiocarcinoma intrahepático multifocal metastásico con mutación BRAF V600E, con el objetivo de ilustrar la relevancia de la caracterización molecular temprana para identificar potenciales dianas terapéuticas en tumores con escasas alternativas farmacológicas.

## CASO CLÍNICO

Varón de 74 años, sin antecedentes familiares oncológicos, hipertenso, dislipémico, exfumador desde los 40 años y consumidor moderado de alcohol. En noviembre de 2019 se le diagnosticó un adenocarcinoma prostático acinar localizado, estadio IIC, que se trató con radioterapia externa (69,6 Gy) y bloqueo androgénico durante dos años, sin evidencia de recidiva.

**Figura 1:**
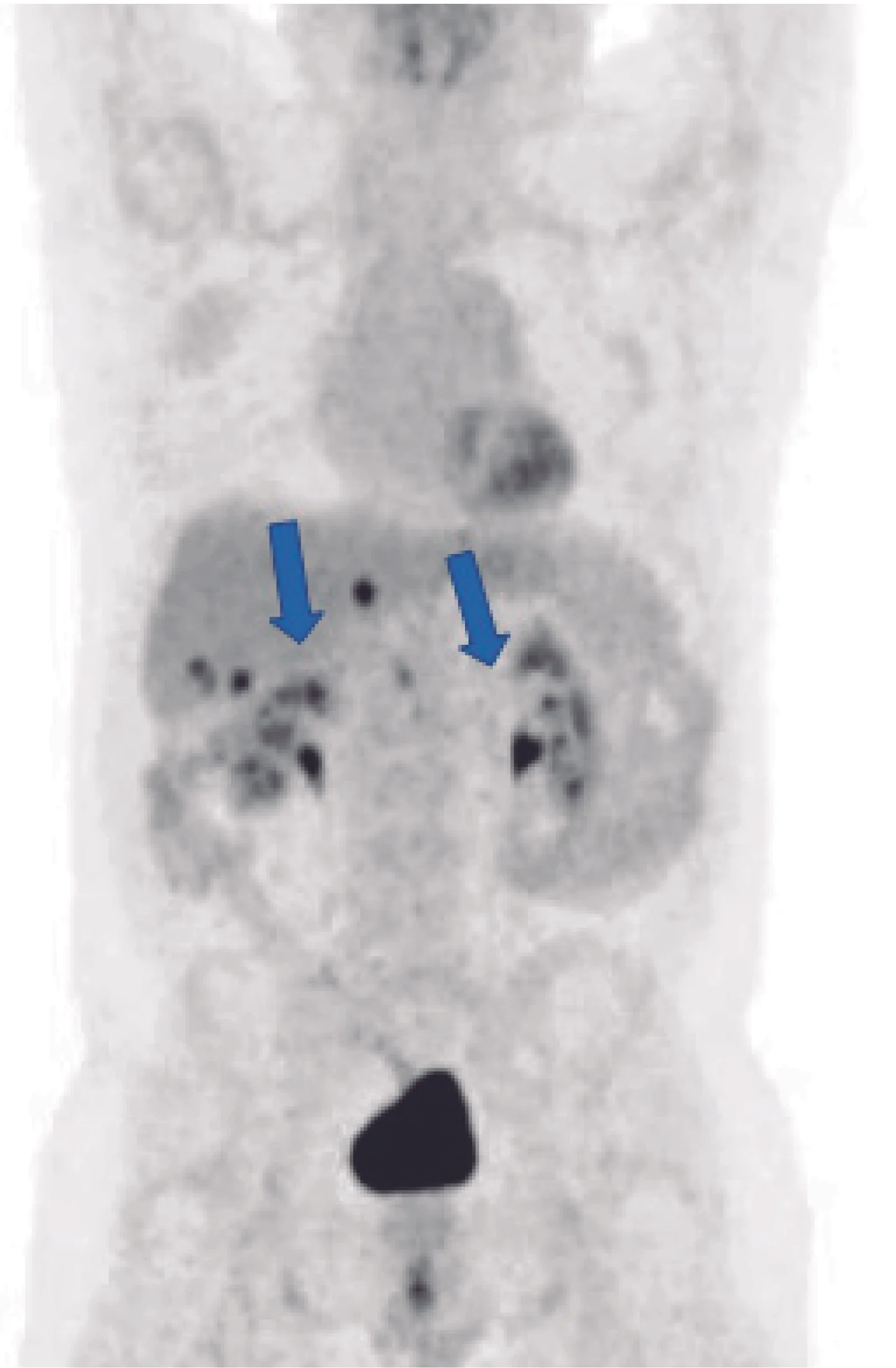
Tomografía por emisión de positrones (PET). Recaída hepática tras cirugía. Se identifican al menos cinco lesiones hipermetabólicas bilobares (flechas azules).

En junio de 2021 se le realizó una resonancia magnética nuclear (RMN) que mostró una lesión en el segmento VI hepático con aumento de tamaño, sugestiva de metástasis. Tras la discusión del caso en el comité de tumores y la realización de una tomografía por emisión de positrones (PET), se decidió derivar al paciente a cirugía con intención curativa, con diagnóstico preoperatorio de CCA intrahepático multifocal con metástasis hepáticas resecables. Se practicó una segmentectomía laparoscópica asociada a hepatectomía parcial.

Posteriormente, el tumor fue reestadificado mediante tomografía axial computarizada (TAC) como T2NxM0 (estadio II) por presentar invasión vascular intrahepática. El paciente recibió quimioterapia adyuvante: ocho ciclos de capecitabina cada 12 horas durante 14 días, en ciclos de 21 días. Los efectos adversos a destacar fueron un síndrome palmo-plantar grado 1 y una toxicidad ungueal leve.

En octubre de 2022 se observó evolución radiológica tras cirugía en el estudio PET, con recaída hepática y ganglionar: cinco lesiones hipermetabólicas bilobares en los segmentos II, V y VII, y un nódulo hipermetabólico en la cabeza pancreática (Fig. 1).

Se le prescribió primera línea de tratamiento con ocho ciclos de cisplatino y gemcitabina (recibidos entre noviembre de 2022 y abril de 2023), dado que al inicio del tratamiento la combinación con inmunoterapia aún no estaba aprobada ni financiada para su utilización en la práctica clínica. En el quinto ciclo se redujo al 80% la dosis de cisplatino por neutropenia grado 3. Según la TAC y el marcador tumoral CA 19-9, la enfermedad se mantuvo estable.

**Figura 2:**
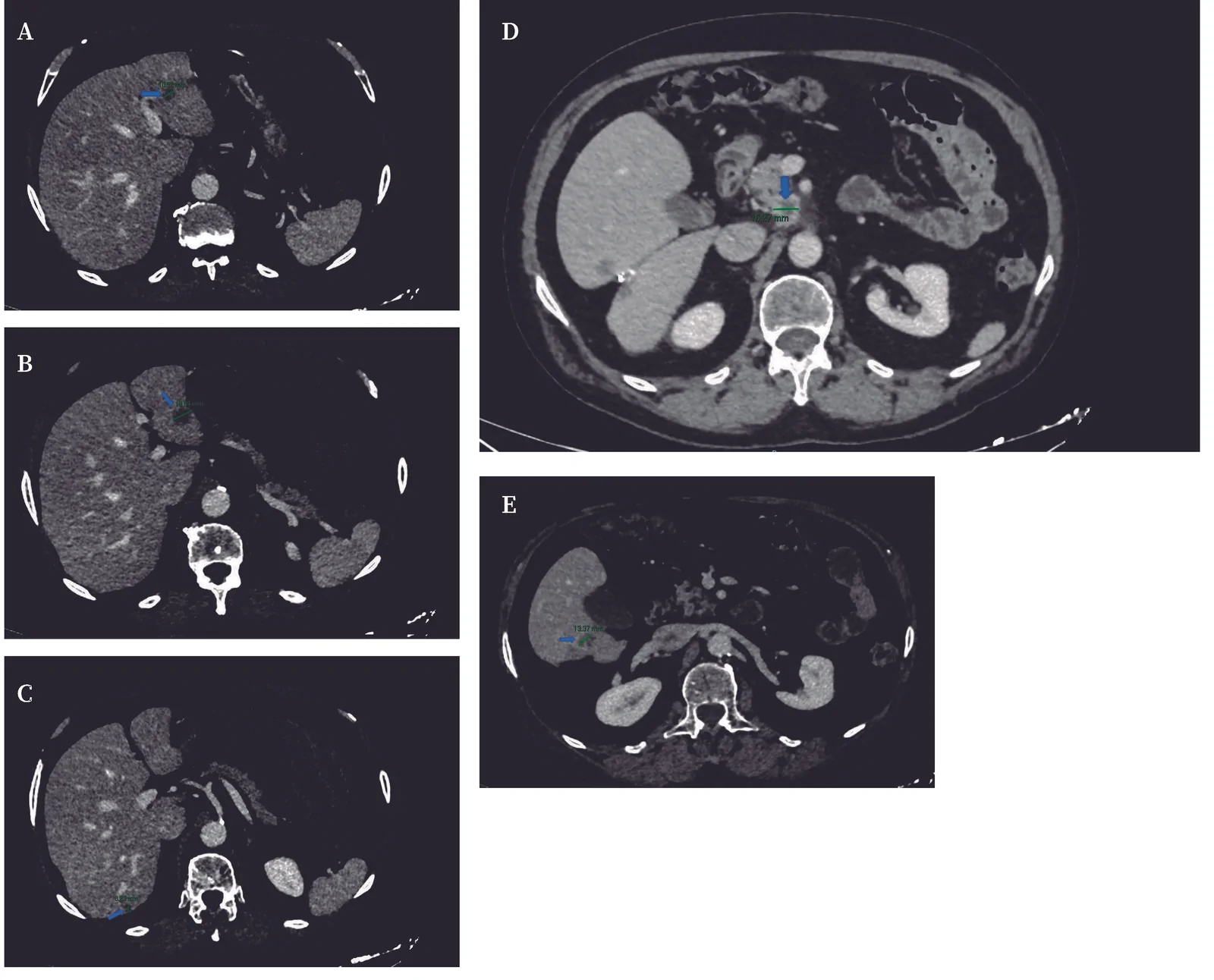
Tomografía axial computarizada (TAC). Lesiones focales y adenopatía necrótica (flechas azules).

En septiembre de 2023, tras referir dolor lumbar y abdominal, se le realizó una TAC que mostró seis lesiones focales con metástasis en los segmentos III, VI y VII, y una adenopatía necrótica de 16 mm cerca de la arteria mesentérica superior, hallazgos compatibles con progresión de la enfermedad (Fig. 2D).

Se solicitó NGS, detectándose una variante patogénica somática c.1799T>A (p. Val600Glu) en el exón 15 del gen BRAF, con frecuencia alélica del 28,76% y relevancia terapéutica con un nivel de evidencia I. Como segunda línea, fuera de indicación, se prescribió, terapia dirigida con dabrafenib 150 mg cada 12 horas y trametinib 2 mg diarios de forma continua hasta toxicidad o progresión.

Hasta finales de octubre de 2024, el paciente se mantuvo con enfermedad estable, con descenso de CA 19-9 y eventos adversos leves como presencia de onicolisis y astenia grado I. La supervivencia libre de progresión (SLP) fue de 12,8 meses,

En noviembre de 2024, la adenopatía tumoral necrótica retroperitoneal mostró un aumento de 1,2 cm en la TAC, con infiltración de estructuras adyacentes. Sin otras mutaciones identificadas por NGS, se le administró un ciclo de FUOX con ajuste de dosis, tras el cual el paciente mostró mal control del dolor (escala visual analógica EVA 10/10) y deterioro clínico. Esto condicionó la suspensión de tratamiento y derivación del paciente a la unidad de Cuidados Paliativos.

El paciente falleció en febrero de 2025 tras una supervivencia global (SG) de 44,4 meses.

## DISCUSIÓN

En este caso describimos nuestra experiencia de uso de la terapia dirigida basada en la inhibición combinada de BRAF y MEK mediante dabrafenib y trametinib contra la mutación BRAF V600E en segunda línea fuera de indicación en un paciente con colangiocarcinoma intrahepático multifocal metastásico y mutación BRAF V600E.

Este abordaje mostró una actividad clínica relevante en nuestro paciente, con una mejoría en la SLP y la SG, además de presentar un perfil de seguridad favorable. Estos hallazgos son consistentes con la evidencia disponible, particularmente con los resultados del ensayo ROAR. El ensayo en fase II ROAR fue el primer estudio prospectivo tipo *basket* que evaluó la combinación de dabrafenib y trametinib en ocho cohortes de pacientes con cánceres raros portadores de la mutación BRAF V600E. La población del estudio estuvo constituida por 206 pacientes, principalmente con leucemia de células pilosas (26,7 %), glioma de alto grado (21,8 %), cáncer de vías biliares (20,8%) y carcinoma anaplásico de tiroides (17,4%). En el conjunto de la población incluida, la combinación de fármacos mostró una mediana de SLP entre 5,5 y 9,5 meses, y una mediana de SG entre 13,5 y 33,9 meses. En el subgrupo de 43 pacientes con cáncer de vía biliar se observaron resultados concordantes: SLP=9,0 meses (IC95%: 5,5-9,4) y SG=13,5 meses (IC95%: 10,4-17,6), junto con un perfil de seguridad manejable. La consistencia del beneficio observado en este subgrupo respalda el uso de la inhibición combinada de BRAF y MEK como estrategia terapéutica en pacientes con colangiocarcinoma y mutación BRAF V600E^9^.

Otra publicación posterior también mostró resultados prometedores del uso de esta terapia combinada en distintos tipos histológicos y localizaciones de tumores con mal pronóstico (SLP=6,5 meses; IC95%: 4,2-7,2 y SG=9,7 meses; IC95%: 7,5-12,2); los efectos adversos más frecuentemente descritos fueron pirexia (26,3%), anemia (12,3%), anorexia (8,8%) e hipoalbuminemia (8,8%)^12^.

En nuestro paciente se consiguió una SLP de 12,8 meses y una SG de 44,4 meses con buena tolerancia, lo que enfatiza la necesidad de utilizar terapias dirigidas a la genómica tumoral para tratar en vida real tumores con escasas opciones terapéuticas. En la práctica clínica, por tanto, las pruebas para detectar mutaciones BRAF V600E deben introducirse en etapas tempranas del plan terapéutico para beneficiarse de nuevas opciones de tratamiento que permitan mejorar la supervivencia.

En conclusión, la terapia dirigida basada en la inhibición combinada de BRAF y MEK mediante dabrafenib y trametinib logró que nuestro paciente con CCA intrahepático metastásico alcanzara cifras de supervivencia superiores a las descritas en la literatura. Es necesario diseñar más estudios que evalúen el uso de estas terapias dirigidas frente a alteraciones genómicas potencialmente accionables en el colangiocarcinoma, caracterizado por su corta supervivencia y opciones de tratamiento limitadas.

## Data Availability

Los autores confirman que los datos que respaldan los hallazgos de este estudio se encuentran disponibles en el caso clínico.
